# Differential Growth and Development of the Upper and Lower Human Thorax

**DOI:** 10.1371/journal.pone.0075128

**Published:** 2013-09-20

**Authors:** Markus Bastir, Daniel García Martínez, Wolfgang Recheis, Alon Barash, Michael Coquerelle, Luis Rios, Ángel Peña-Melián, Francisco García Río, Paul O’Higgins

**Affiliations:** 1 Paleoanthropology Group, Museo Nacional de Ciencias Naturales (CSIC), Madrid, Spain; 2 Facultad de Ciencias, Universidad Autónoma de Madrid, Madrid, Spain; 3 Department of Radiology, Medizinische Universität Innsbruck, Innsbruck, Austria; 4 Faculty of Medicine, Galilee Bar Ilan University, Zefat, Israel; 5 Department of Anatomy and Anthropology, Sackler Faculty of Medicine, Tel Aviv, Israel; 6 Fundación Aranzadi, San Sebastián, Spain; 7 Departamento de Anatomía y Embriología, Universidad Complutense Madrid, Madrid, Spain; 8 Hospital Universitario La Paz, Biomedical Research Institute (IdiPAZ), Madrid, Spain; 9 Hull York Medical School (HYMS), University of York, York, United Kingdom; Museo Nazionale Preistorico Etnografico 'L. Pigorini', Italy

## Abstract

The difficulties in quantifying the 3D form and spatial relationships of the skeletal components of the ribcage present a barrier to studies of the growth of the thoracic skeleton. Thus, most studies to date have relied on traditional measurements such as distances and indices from single or few ribs. It is currently known that adult-like thoracic shape is achieved early, by the end of the second postnatal year, with the circular cross-section of the newborn thorax transforming into the ovoid shape of adults; and that the ribs become inclined such that their anterior borders come to lie inferior to their posterior. Here we present a study that revisits growth changes using geometric morphometrics applied to extensive landmark data taken from the ribcage. We digitized 402 (semi) landmarks on 3D reconstructions to assess growth changes in 27 computed tomography-scanned modern humans representing newborns to adults of both sexes. Our analyses show a curved ontogenetic trajectory, resulting from different ontogenetic growth allometries of upper and lower thoracic units. Adult thoracic morphology is achieved later than predicted, by diverse modifications in different anatomical regions during different ontogenetic stages. Besides a marked increase in antero-posterior dimensions, there is an increase in medio-lateral dimensions of the upper thorax, relative to the lower thorax. This transforms the pyramidal infant thorax into the barrel-shaped one of adults. Rib descent is produced by complex changes in 3D curvature. Developmental differences between upper and lower thoracic regions relate to differential timings and rates of maturation of the respiratory and digestive systems, the spine and the locomotor system. Our findings are relevant to understanding how changes in the relative rates of growth of these systems and structures impacted on the development and evolution of modern human body shape.

## Introduction

The thoracic skeleton is an osteo-cartilaginous framework that surrounds and protects the thoracic viscera and supports the mechanical function of ventilation. To fulfill its role in ventilation the thoracic skeleton offers a large surface area for muscle attachment (intercostal muscles, diaphragm, and accessory respiratory muscles) [1,2]. The muscles act to raise the ribs, which increases thoracic dimensions as a consequence of their angulation, form and joints. This leads to reduced intra thoracic pressure and so, to inspiration [1,2]. Expiration, as the ribs return to their original positions, is more passive. Thus, chest wall dynamics depend on rib morphology [1]. In consequence, how morphology changes postnatally is relevant clinically as well as to physiological modelling, and functional and evolutionary morphology [2-19].

We as yet have a poor understanding of how the thorax grows in size and develops in form throughout life. Although the advent of 3D imaging means that CT scans of the thoracic wall and contents are readily available [20-22], a major problem has inhibited the characterization of thoracic ontogeny: the difficulty in quantifying the detail of the 3D-features of thoracic wall and rib-curvature and the spatial relations of the ribs, sternum and spine. Despite these difficulties, one 3D geometric morphometric analysis has examined changes with old age in adult male ribcage morphology [11], demonstrating widening in the lower thorax but providing no information on variations in rib curvature. Kagaya and colleagues [23] assessed rib curvature and thoracic shape in anthropoids using Bezier curves fitted to alternate ribs. Their data discriminated between the barrel-shaped thorax of *Hylobates* and the funnel-shaped thoraces of other hominoids (but see 24 and Discussion)

No 3D geometric morphometric analysis has yet addressed ontogenetic variation in humans. To date, ontogenetic studies of the human thorax have used linear distances and indices computed from them to describe the principal dimensions of the thorax or of a few ribs [25,26]. One such study [25] concluded, from analysis of indices of averaged linear measurements in horizontal cross sections at the manubrio-sternal junction, the diaphragmatic dome and midway between these reference levels, that adult-like thoracic shape is achieved early, by the end of the second postnatal year. In achieving this transformation from the circular cross-section of the newborn into the ovoid section of adults, the ribs become inclined such that their anterior borders are inferior to their posterior ones.

A key aim of the present study is to revisit the ontogeny of thoracic form to obtain a fuller picture of how it changes throughout life. This is because there are good reasons to expect a more complex picture of human thoracic ontogeny than that described above. Specifically, different ontogenetic changes can be expected with regard to upper (ribs 1-5) and lower (ribs 6-10) thoracic morphology (see Methods) since these regions are related to different organ and body systems that mature differentially during ontogeny. Thus, the upper thoracic region is related to the pulmonary part of the respiratory system and the upper limbs [27] while the lower thorax is anatomically related to the diaphragmatic part of the respiratory system, and also more closely to the abdominal cavity and locomotor apparatus [5,16]. Additionally, continuous descent of the anterior parts of the ribs is part of the aging process, related to a decline in lung function and vital capacity [11]. Finally, secondary ossification centres appear close to the articular tubercles around puberty [28,29] suggesting that new features of ontogenetic shape change may appear during later ontogeny.

Openshaw et al. [25] focused on the mid-thorax and as such, our knowledge of the ontogeny of ribcage form above and below this level is limited. No study has yet examined the detail of whole ribcage postnatal ontogeny and its spatial and temporal associations with the growth and maturation of related body systems. A useful analogy might be drawn with the craniofacial skeleton, which comprises modules that show a degree of independent growth that is reflected in curved ontogenetic trajectories of shape change [30-32]. Likewise, in this study we test the hypothesis that upper and lower thorax behave as modules with a degree of independence in ontogenetic trajectories.

To these ends we apply geometric morphometric methods to extensive 3D landmark configurations to assess how thoracic form and the form and spatial relationships of the ribs and sternum covary with ontogeny and the extent to which upper and lower regions of the thoracic covary throughout postnatal ontogeny. Particularly, we test the hypothesis that directions of shape change differ in the upper and lower parts of the thorax. This represents the first such study in humans.

## Methods

Computed tomography (CT) data were obtained from subjects that were scanned previously for medical reasons unrelated to this study. All patients were scanned in supine position in maximum inspiration (Austria) except three newborns scanned in France, where two subjects were scanned for trauma in unknown respiratory status and one subject was scanned for virtual autopsy post mortem. However, in none of the cases any obvious pathologies affected skeletal thoracic form. The age and sex composition of the sample is detailed in Table 1 (N=27). Because the subjects were scanned previously for medical reasons unrelated to this study (retrospective), it is lawful and not necessary to obtain consent from the next of kin, caretakers, or guardians on the behalf of minors/children participants of this study. Consequently such consent was not required by the local ethic committees following local laws. The approval to use these pre-existing Ct scans for our research was obtained in writing from the *Comiteconsulitatif* *pour la protection des personnes dans la recherche biomédicale Bordeaux A* and from the Ethikkommission der Medizinischen Universität Innsbruck (AN5025, 323/4.24) (copies of approvals of both ethics committees have been submitted to manuscript central). Prior to analysis all CT-data were anonymized to comply with the Helsinki declaration [33].

**Table 1:** CT-data sets, sex and ages (age group definitions in Material and Methods).

**Id**	**Age (years**)	**sex**	**age group**
TX001	1	female	group1
TX002	3	female	group2
TX003	6	female	group2
TX004	11	female	group2
TX005	14	female	group2
TX006	40	male	group3
TX007	60	male	group3
TX008	50	male	group3
TX010	62	female	group3
TX011	27	female	group3
TX012	59	male	group3
TX013	0.08	male	group1
TX014	0.25	female	group1
TX015	0.4	male	group1
TX022	0.11	male	group1
TX024	18	male	group3
TX025	6	male	group2
TX026	7	male	group2
TX027	8	male	group2
TX028	10	male	group2
TX029	0.06	male	group1
TX030	0.6	male	group1
TX031	1.8	male	group1
TX032	4	male	group2
TX041	15.6	female	group2
TX045	5	male	group2
TX046	10.5	female	group2

### Sliding semilandmarks

**Figure 1:**
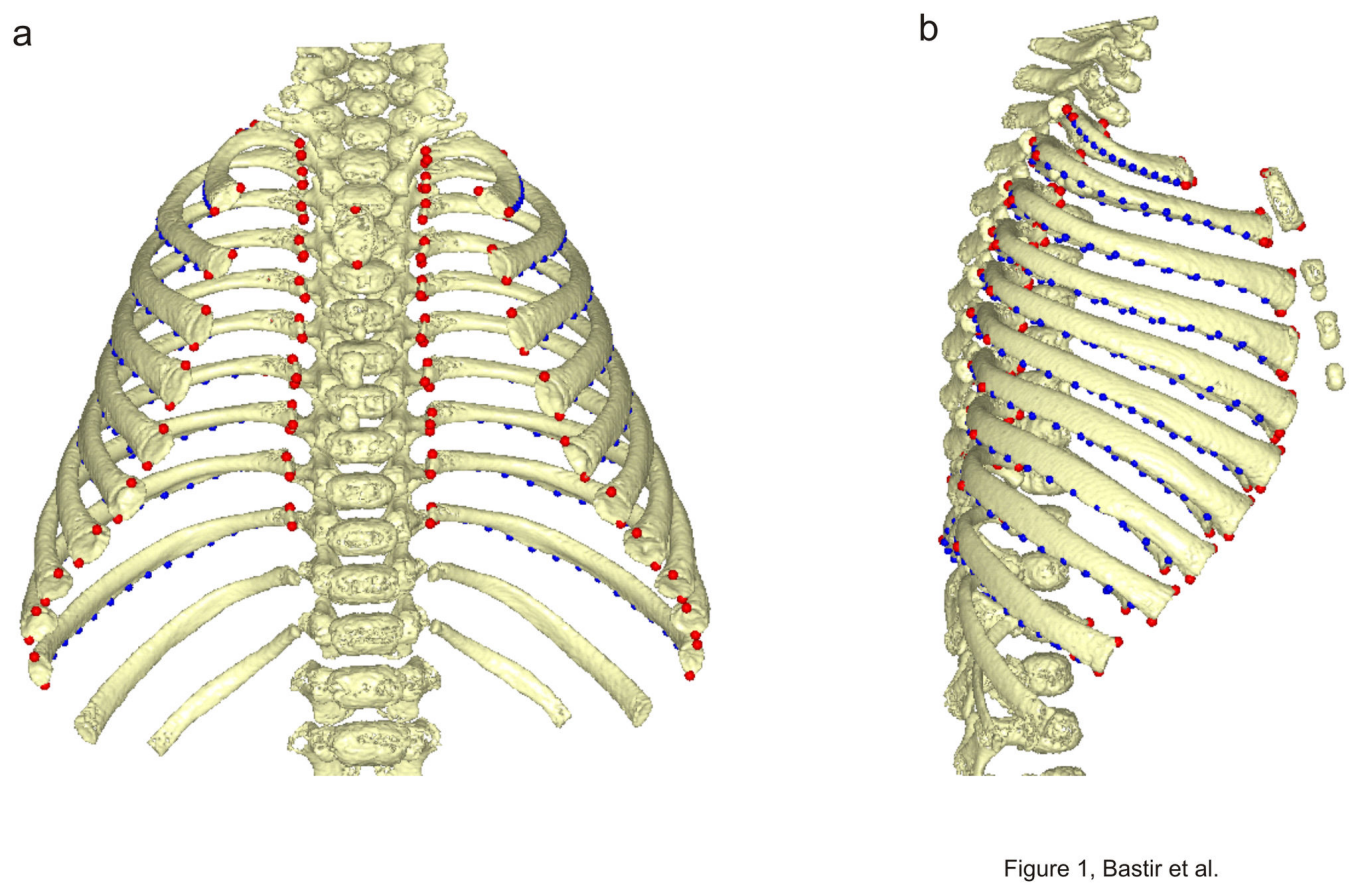
3D landmarks. Landmarks (red) and sliding semilandmarks (blue) used to describe the thoracic skeleton; newborn subject in frontal (a) and lateral (b) view.

Landmarks and semilandmarks for sliding [34] were located on skeletal elements in-situ within 3D CT based reconstructions of the thorax. As such, landmark subsets describe the form of individual skeletal elements while the full landmark configuration describes the form of these elements, their relations to each other and the overall form of the thorax. On the ribs, landmarks were placed at the most superior, anterior and inferior points of the head, the most lateral point of the articular tubercle, the most inferior point at the angle at the lower rib border, (where the angle is most doubtlessly recognizable) and the most superior and inferior sternal extremes. Additionally 15 equidistant semilandmarks were sited along the lower costal border between the articular tubercle and the inferior sternal extreme. Each rib was thus described by twenty 3D landmarks. At the sternum two landmarks were sited in the midline, one in the manubrial notch and the other on the inferior border. The full data comprise 402 landmarks and semilandmarks (Figure 1).

The surface of the bony structures of the rib cage was segmented with threshold based techniques following the “full width half maximum” approach [35] thus allowing for reproducible results. We used this protocol in Amira 4 software (www.vsg3d.com) and obtained reasonably well represented 3D models of bony structures, which were further post-processed (cleaning, smoothing, mesh hole-filling, standardized positioning) by Artec Studio software (www.Artec3D.com) [36-39]. Final 3D models were then imported into Viewbox4 software (www.dhal.com) to position 3D landmarks and semilandmarks along the inferior curves of ribs 1-10. Because of uncertainty in terms of their locations along the ribs, semilandmarks were then slid along their corresponding curves with respect to the fixed landmarks so as to minimize bending energy, first during landmarking between each specimen and the template (first specimen) and after that, a second time against the sample average configuration [30,34]. This procedure adjusts their relative locations along the curve. After sliding the semilandmarks on the lower border of each rib represent the shape of the lower border. The semilandmark set for each curve should be interpreted as a whole, i.e. as a single curve, rather than as discrete points. We also used a TPS approach with the semilandmarks to estimate missing data, which in a few cases was necessary for the landmarks at the sternal extremes of ribs 9 and 10 [37,38,40]. Finally, the resulting 32562 3D-measurements (x,y,z-coordinates) were analyzed statistically.

### Age groups, statistical analyses and visualization

In order to address Openshaw et al.’s [25] hypothesis, that adult-like thoracic shape is achieved by the end of the second postnatal year the data were divided into three groups. Group 1 contained individuals ranging from newborns to two years following Openshaw et al.’s [25] hypothesis. Group 3 was composed of adults (above 18 years [30]). Group 2 contained all subjects between from three to eighteen years. Despite its large range of ages, group 2 is potentially interesting due a considerable lack of knowledge about respiratory apparatus ontogeny in these stages [41,42].

Shape data were symmetrized in MorphoJ-software [43] using reflected relabelling and principal components analysis (PCA) of Procrustes shape coordinates was carried out to visualise ontogenetic shape trajectories in PC1-2 and PC1-3 projection. We also used these PCA projections to assess how many non-adult individuals plot within the 95% confidence intervals of adult thoracic shape configurations. To optimally investigate full thoracic growth allometry we also performed a PCA in Procrustes form space [40,44,45].

In addition to the PCA analyses in shape and form spaces we used the age groups to compare mean shapes and mean sizes (centroid size) to further evaluate the hypotheses. Mean shapes were compared by permutation tests of group membership (N=10000) [40,46]. Centroid size followed a normal distribution (KS d=0.135, p=n.s) so ANOVA and Bonferroni post-hoc comparisons were used for group mean size comparisons [47].

Our data come from anonymized clinical hospital CT scans and visual inspection did not reveal signs of skeletal morphological alterations due to pathology. However, to validate quantitatively our visual assessment of normal skeletal morphology, a control group of CT data of four healthy adults related to different (and not yet published) research was compared with the six adults of the present study. Our validation analysis showed a complete overlap in principal components space of the known-healthy adults and the adults in this study. Also, mean shape comparisons did not produce statistically significant results. Consequently, normal skeletal morphology is assumed for the full sample.

### Upper versus lower thorax ontogeny

To test the hypothesis that the upper and lower thorax follow different ontogenetic trajectories [48-50], the landmark data were divided into an upper (ribs 1-5) and lower part (ribs 6-10). Such a division reflects the fact that the upper part is more related to respiration, upper limb articulation and movement, while the lower part is more related to diaphragmatic respiration, posture, and subthoracic viscera (e.g. intestines, liver, reproductive systems) [1,2,5,6]. Many of these systems grow with different ontogenetic maturation patterns.

Because both parts share the same number of landmarks a common superimposition was possible, placing them into the same shape space. Therefore, to compare their ontogenetic allometries multivariate regressions of shape on centroid size were computed for each and the angle between these regression lines was calculated. Small angles indicate similar, and large angles different relationships between shape and centroid size [49,51]. Statistical assessment of the resulting angles is often done by comparison of true angles to those between randomly permuted groups in the multivariate space of interest [48,50]. Random vectors are drawn from a uniform distribution on a hypersphere with the appropriate dimensionality. These comparisons have usually been done by randomisation, but a closed-form formula for the probability has recently been published [52] which allows rapid computation of significance levels [51]. The angles were computed and assessed statistically using MorphoJ software [43].

### Hypothesis testing

Openshaw et al’s[25]. hypothesis would be supported if all individuals older than 2 years (groups 2 and 3) plot within the 95% range of the adults. Additionally, the hypothesis predicts significantly different mean shapes between group 1 and group 2, group 1 and group 3 and (less to) no difference between group 2 and group 3.

The hypothesis of modular growth of the upper and lower thorax is assessed by computing the angle and its significance between the multivariate regressions of shape on centroid size for these regions. Non-significant angles indicate a lack of evidence for significant differences, small but significant angles indicate similarities, and large significant angles indicate differences.

In addition to the angle comparisons, visual inspection of 3D warped thoracic surfaces along the first principal component of form space, which reflects the majority of growth allometry [44,45], allowed appraisal of ontogenetic differences in the upper and lower thorax. The surface warps are based on thin-plate splines and thus contain measured information at the landmarks and semilandmarks and interpolated information (not directly measured) at the remaining parts of the surface meshes.

## Results

### Size analysis

**Figure 2:**
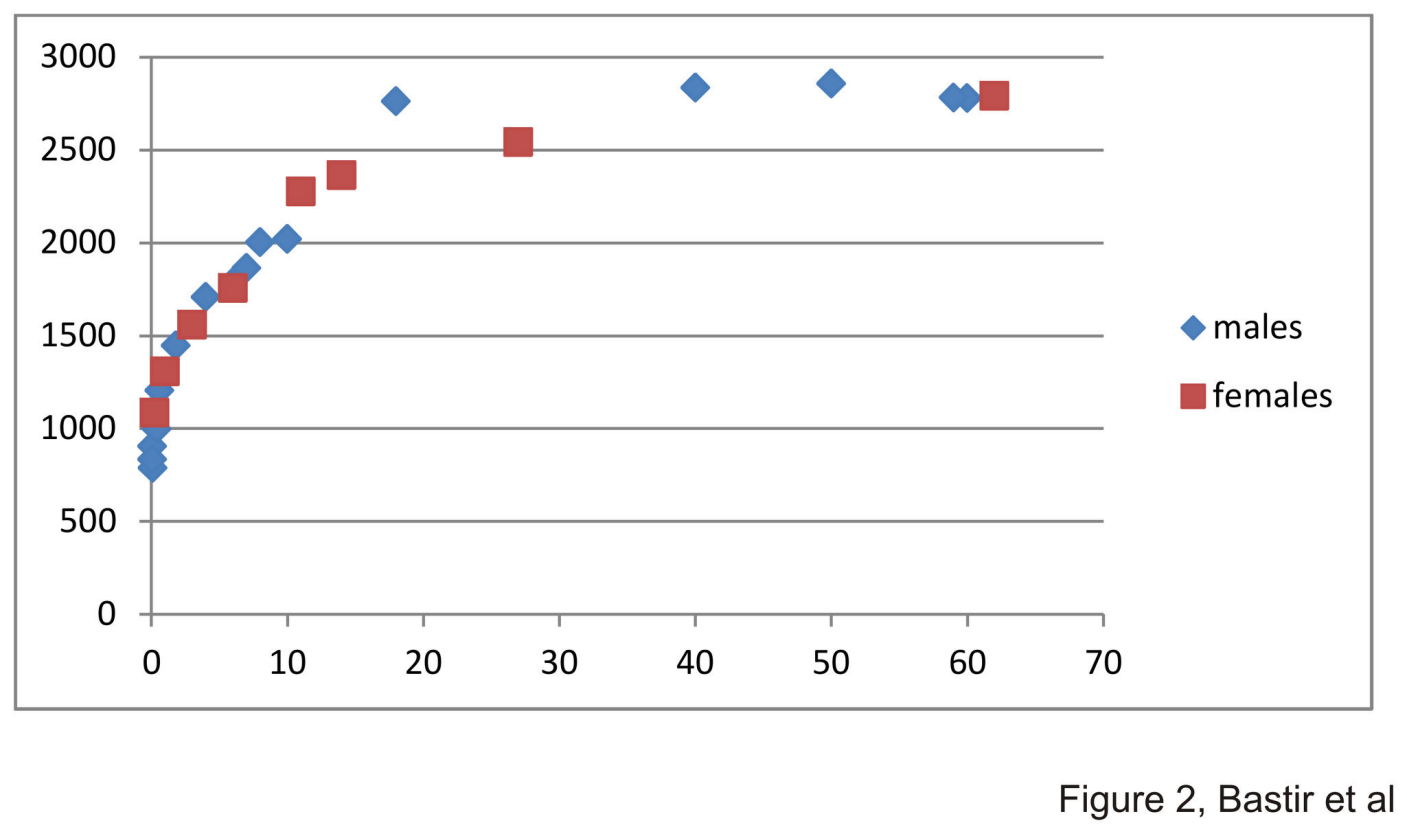
Ontogenetic increase of thoracic size. Changes in centroid size in males and females. X-axis shows age in years, y-axis shows centroid size.

Growth is characterized by a high rate of increase in centroid size in the early years, tailing off slowly until a marked deceleration occurs at around 10-11 years. Figure 2 suggests that adult thoracic size is achieved in adolescence around 14-15 years. ANOVA revealed highly significantly different group means (F(2, 25)=115.89; p=0.0001;CS of group 1: 1072.5; CS of group 2: 1951.4; and CS of group 3: 2761. Bonferroni post-hoc corrections identified highly significant differences in all pairwise comparisons (between MS=4928; df=25; p=0.001).

### Shape analysis

**Figure 3:**
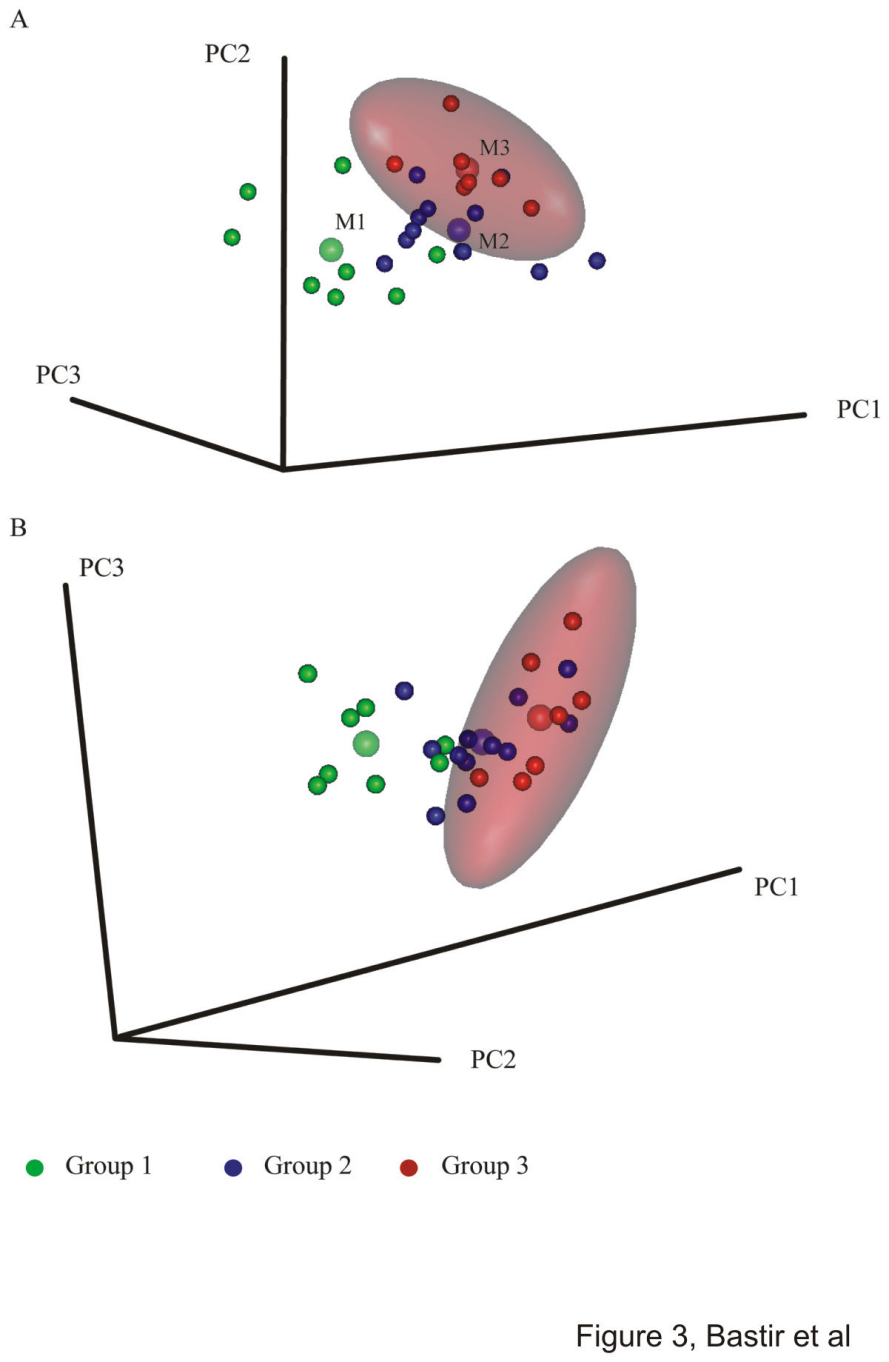
Principal components analysis in shape space. 3D Scatterplots of principal components of shape with 95% confidence intervals of the adults (red ellipses) (a) PC1 versus PC2, (b) PC1 versus PC3. Note that 95% of the adult range in (a) excludes not only all group 1 subjects but also almost half of group 2. (b) The adult range includes most of group 2 and excludes most of group 1. Group mean markers are slightly enlarged and semitransparent.

The 95% confidence intervals (CI) in the PCA of shape space (Figure 3) suggest that the hypothesis of Openshaw et al [25] is not fully supported in terms of shape. However, this depends to some degree on the projection. In the PC1-2 subspace of shape (PC1: 52.34% of total variance, PC2: 13.67% tot. var.) the 95% CI includes only half of the individuals of group 2 leaving out six individuals aged older than 2 years. All individuals of group 1 are outside the adult 95% range. However, in the PC1-PC3 subspace (PC3: 10.23% tot. var.), all but two subjects of group 2 and two members of group 1 are included within the adult range. Taking both plots together adult shape appears to be the result of both early and later ontogenetic modifications.

### Procrustes form space analysis

**Figure 4:**
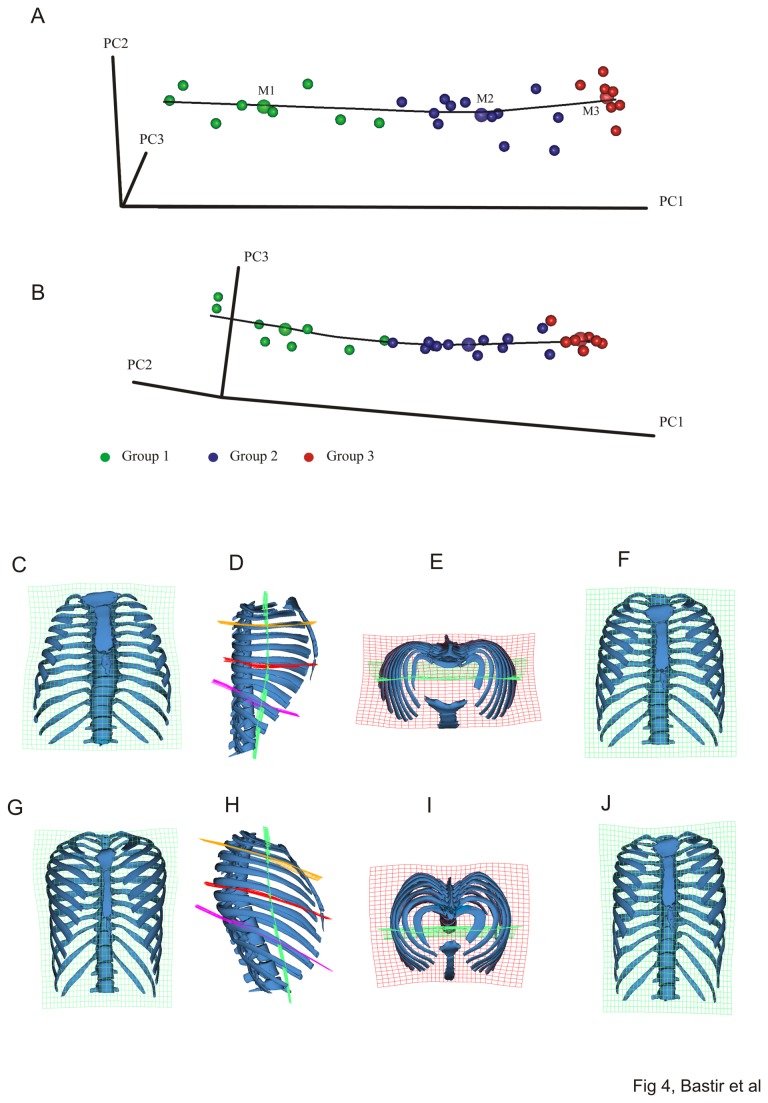
Principal components analysis in Procrustes form space. Form space ontogenetic shape trajectory. The growth allometry is curved in form space. A line between the enlarged semitransparent dots representing the means of the three age groups illustrates this change of orientation in growth allometry. Different projections of the ontogenetic shape trajectory show (A) PC1-PC2 and (B) PC1-PC3. The warped ribcage models show frontal views of the smallest (youngest) specimen (C) and the largest individual (G). The green transformation grid (x-y plane) shows relative upper thoracic expansion and lower thorax contraction during growth from C to G. The lateral views show shape changes from the smallest (D) to the largest individual (H) and demonstrate the complex changes in rib orientation, axial and lateral curvature. These ae more pronounced in the upper thorax (orange and red TPS grid) than in the lower (violet TPS grid) during growth. Note how the lower thoracic spine and the relative elongation of the lower ribs both contribute the lower thoracic shape changes. Superior views show the realtively strongly medio-laterally expanded thorax of the smallest (E) and the realtivley deeper chest in the largest (I). This view also shows that the posterior-most structure in the smallest individuals is the spine while in the largest it is the bilateral posterior projection of the ribcage lateral to the *angulus* *costae* (invagination of the vertebral spine). The frontal views in F) and J) illustrate the changes in thoracic shape represented by PC3 which are a considerable component of the differences between the means of group 2 (blue) and group 3 (red). These changes likely reflect growth of stature during later ontogeny.

PCA suggests a very tight association between size and shape and also shows the age groups are well-ordered along PC1 (95.9% of total variance, PC2: 1.3% and PC3: 0.7%). A plot of PCs1, 2 and 3 (98% of total variance) suggests a gently curved allometric growth trajectory (Figure 4). Between group 2 and group 3 the orientation of the trajectory changes with particularly with changes along PC3 adding to the general growth changes along PC1.

The warps associated with PC1 in form space (Figure 4C-I, Movie S1) show that the newborn thorax is pyramidal, with a narrow upper and a wider lower medio-lateral diameter (Figure 4C). The ribs of the upper thorax in newborns are mostly horizontal, whereas the ribs of the lower thorax are downwardly inclined (Figure 4D). After growth, all ribs show a sternal elongation, and a downward curve at the sternal ends (Figure 4H), but, in addition to the rib elongation, the sternal ends of the lower thorax also become shifted anteriorly due to increased lumbar curvature of the spine (Figure 4H).

The growth expansion of the upper thorax (Figure 4G) is produced by a complex curvature change in which the mid-third of the rib shaft not only expands medio-laterally but also curves upwards, relative to its costo-vertebral attachments and the sternal extremes. Figure 4H shows a lowering of the rib orientation. A top view shows a considerably “invagination” of the spine (Figure 4I). As a consequence of that process in the smallest and youngest the posterior-most structures of the thorax are the spinous processes of the thoracic vertebrae in the midline, while in adults themost posterior structures are parts of the rib cage located lateral to the *angulus costae* (compare posterior thorax outline in Figure 4E and 4I).

As a consequence of all these changes the upper thorax starts off in newborns with a circular and ends up, in adults, with an ovoid cross section, which fits with the prediction of Openshaw et al. [25]. However, the lower thorax starts with an ovoid cross section and ends up with a circular one in adults, which is contrary to the prediction (see mean shape comparisons below).

Statistical comparisons of the three age group means (Table 2) show clear differences between group1 and group 2 and between group 1 and group 3, which differ at p<0.0001 with 10000 permutations. The significance of mean shape differences between group 2 and group 3 varied between between p<0.04 and p<0.08 during different randomization analyses.

**Table 2:** Mean shape comparisons (Procrustes distance, p-levels).

	**Group 1**	**Group 2**
**Group 2**	0.111 (p<0.0001)	
**Group 3**	0.1375 (p<0.0001)	0.0564 (p<0.045-0.081)

### Quantified morphological features of mean shapes

**Figure 5:**
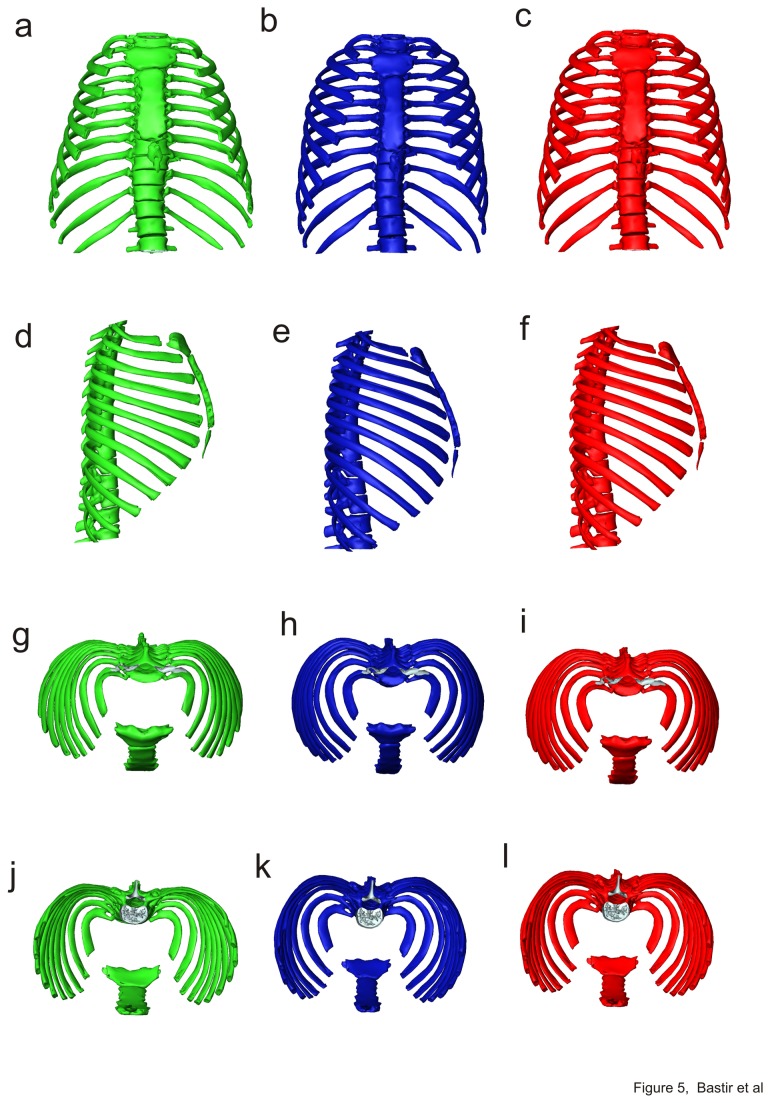
Mean shapes of age groups. Group 1 (green), 2 (blue) and 3 (red) in frontal (a-b), left lateral (d–f), superior (g-i) and inferior (j-l) views.

**Figure 6:**
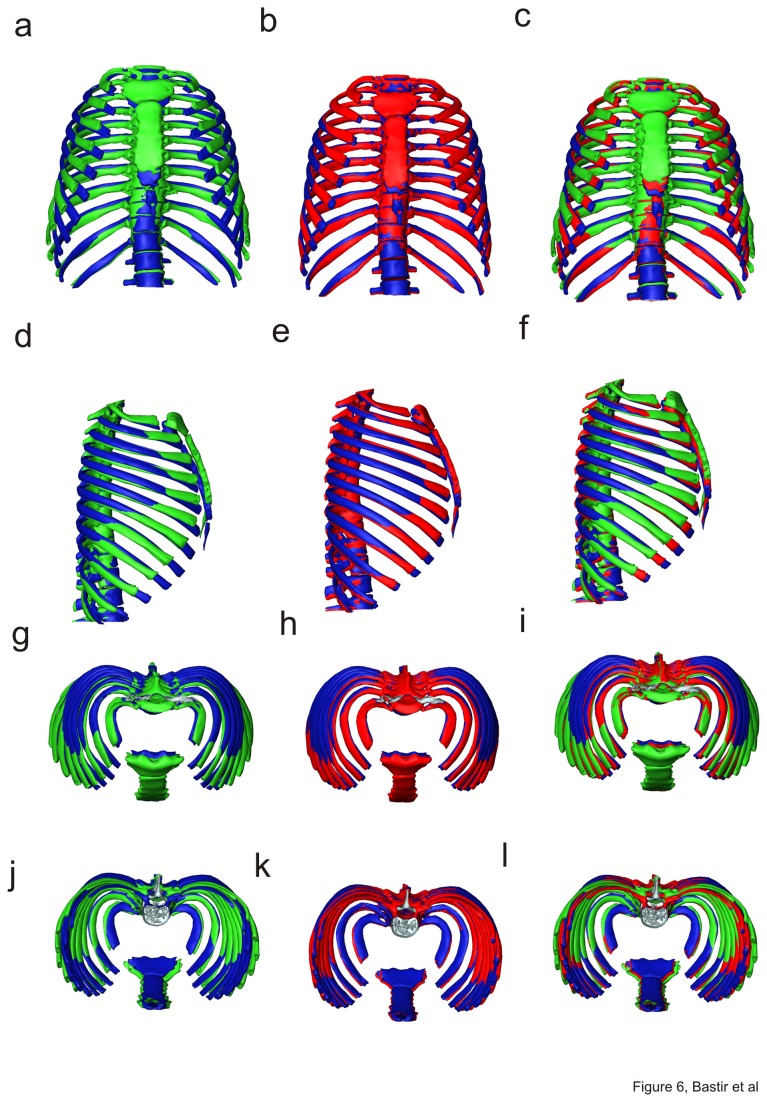
Procrustes registered means of age groups. Group 1 (green) and 2 (blue) (a,d,g,j), age groups 2 (blue) and 3 (red) (b,e,h,k), and of all three age groups in frontal (a-b), left lateral (d–f), superior (g-i) and inferior (j-l) views.

Mean shapes for each age group are shown in Figure 5, and are superimposed for comparison in Figure 6. The first phase of growth between age groups 1 and 2 (Figure 6; a, d, g, j) results in an increase of the upper thorax (Figure 6a, d, g, j) relative to the lower thorax which becomes narrower (Figure 6a, j, compare also with PC1 warps in Figure 4c, g, and Movie S1). This produces a more barrel shaped frontal outline (Figure 5a vs. 5b). Relatively more posterior positioning of the angles of the upper ribs (Figure 6d) deepens the posterior parasagittal guttering of the rib cage. The physiological kyphosis of the thoracic vertebral column (Figure 5d, vs. 5e) also increases. Further, the lateral (Figure 5d, e, Figure 6d, e) and axial views (Figure 5g, h; Figure 6g, h) show that the antero-posterior dimensions decrease relative to lateral ones in the midline (Figure 5h, j) while the sternal portions of the ribs expand anteriorly (Figure 6d,g). As a consequence, the upper part of the sternum shifts posteriorly and becomes lowered in age group 2 when compared to age group 1 (Figure 6d). This is accompanied by a relative decrease in thoracic antero-posterior diameter (Figure 6g, j) despite relative anterior lengthening of the upper ribs. Increased antero-posterior angulation of the upper ribs (attaching to the sternum; Figure 6a, d) relative to the spine accompanies and likely contributes to the descent of the sternum and the consequent relative decrease in upper thoracic antero-posterior diameter. The posterior part of the upper thorax becomes elevated relative to the anterior due to lateral elevation (Figure 6d). At the same time, the rib angles project more posteriorly, which increases lateral antero-posterior thoracic diameters relative to the midline (Figure 6g, h). In consequence, in group 1 the maximal antero-posterior dimension of the thorax is at the mid-sagittal plane (Figure 5g), while in group 2 the largest a-p diameters are found bilaterally, off the midline, easily appreciated in axial view (Figure 5h) (see also Figure 4e, i). This is also related to the development of the physiological lordosis in the thoracic part of the vertebral column which is relatively straight in group 1 and develops later (Figure 5, d,e).

Between age groups 2 and 3, in the second growth phase (compare Figure 4f, j) changes in thoracic shape are less pronounced (Figure 5; Figure 6, b, e, h, k) and in contrast to the transformation between age groups 1 and 2 they are concentrated in the mid- and lower thorax. This change results in more subtle differences that lead to a small relative increase in upper thoracic height (Figure 6e, see also Figure 4f,j) and in lower anterior thoracic width (Figure 6b,h,k). The upper thorax slightly increases in a-p diameter and this is (Figure 6e), accompanied by a relative forward shift of the sternum (Figure 6e,h, k).

### Upper and lower thoracic growth vectors

Multivariate regressions of shape on size of the upper thorax explained 36.4% (p<0.001), and of the lower thorax 42.9% (P<0.001) of the total variance in thoracic shape. The angle between regression lines was highly statistically significant (p<0.0001) at 36.4 degrees. This result supports the hypothesis of different growth allometries for the upper and lower thorax.

Taking together the ontogeny of centroid size and the shape and form space trajectories, our findings suggest the existence of significant changes in form between the three age groups.

## Discussion

This study has examined how the thorax changes in shape, and size over time and has tested the hypothesis that adult thoracic shape is achieved early, by the age of two [25]. Beyond this, it has been possible to compare the growth allometries of the upper and lower thorax and so to consider, below, how these integrate respiratory function with the demands of locomotion (posture) and the relative size of the abdominal contents, principally the digestive system.

The analyses of this study indicate that postnatal ontogenetic shape changes in the thorax are complex and show a shift between early and later phases of ontogeny. Consistent with the hypothesis [25], the early growth phase comprises relatively marked changes in thoracic shape between birth and the third year of postnatal life, particularly in the transverse diameter of the upper thorax relative to the lower [25,53]. Thus, Figure 6 shows that by the third year of life the pyramidal neonatal thorax is transformed into the more barrel shaped thorax typical of adults. Our analyses also demonstrate that further, but smaller ontogenetic changes in shape and particularly size later fully establish the typical adult rib cage configuration.

Our results show that widening in the coronal plane is particularly a feature of upper thoracic ontogeny (3^rd^ to 5^th^ rib) (Figure 5). This is likely integrated with the growth of the lungs, which show a major increase in volume during the first two years [54]. In contrast, the lower thorax of the newborn is relatively wider than that of adults. This contrasts with Openshaw et al.’s [25] findings (from midsternal transverse sections) and likely relates to relative growth differences between respiratory and digestive (including the liver) systems as well as relative vertical lengthening of the abdomen, such that the abdominal contents can be accommodated by a relatively narrower abdomen. Thus, the transformation from a pyramidal to a barrel shaped thorax is reminiscent of the difference between great apes and modern humans, where the former have a pyramidal thorax, at least in part to accommodate the relatively greater volume of the abdominal organs. In this case the differences in shape are driven by dietary differences [55] whereas in human ontogeny they are driven by differences in developmental trajectories between thoracic and sub-thoracic organs and spines. Thus, very different anatomical factors can lead to pyramidal thoracic shapes with narrower upper and wider lower openings. This has recently been indicated by García-Martínez et al. [24]. These authors demonstrated overall morphological similarity in different non-human primate thoraces on the one hand, but with clearly recognizable differences in 3D details of rib curvatures on the other. It has thus been suggested [24] that describing pyramidal versus barrel shaped thorax morphologies in considerations of human and primate evolution leads to oversimplification.

In addition to widening of the upper thorax, the lower sternum becomes relatively elongated and the anterior ends of ribs 2-10 come to lie relatively more medially and posteriorly. Sternal rib orientation is also altered, with the anterior ends coming to lie relatively more inferiorly as the sternum comes to lie relatively more inferiorly. This is due in part to lengthening of the ribs, but the change in orientation of the ribs is also accompanied by increased antero-posterior angulation of the upper ribs and elevation of the posterior third of the rib shaft close to the angle. As a result a complex axial and lateral 3D curvature develops in the upper ribs [56]. This reconfiguration of rib-curvature and orientation is functionally important as it underlies changes in respiratory mode in the first few years of life [57,58]. Breathing in the newborn child is essentially diaphragmatic, and because the ribs are more horizontal than in adults, the respiratory muscles cannot raise them effectively [1]. However, with changing rib-orientation during the first three years, biomechanically more efficient thoracic breathing becomes possible [1,2].

The female adult sternum is located at the level of the third thoracic vertebra while the male is located higher [29,59]. Bellemare et al [60] have linked the lower female sternal position and greater rib inclination to functional adaptations to pregnancy and the female’s capacity to achieve relatively greater volume expansions. Our analyses are unable to determine if lowering of the anterior part of the thorax relative to the posterior (Figure 6d) occurs differentially in males and females or if differences are already present at birth. Larger, sexed, developmental series are necessary to address this.

The development of the thoracic kyphosis contributes to further anterior extension of the sternal rib extremes of the lower sternal ribs (Figure 6) [53,61]. An important interaction between posture, ribcage configuration and respiratory physiology has also recently been identified by a kinematic study [16]. These authors show that malformations of the spine cause inefficient positioning and orientation of the ribs, which precludes the respiratory muscles from coordinated and efficient kinematics [16].

During later childhood and adolescence further, smaller, but important modifications in thoracic shape occur. Centroid size increases steeply (Figure 2) until adult size is reached around adolescence. Little is known about later ontogenetic growth but form space analysis clearly points to an increase in vertical thoracic height (Figure 4f, j), likely in relation to increase in stature [62]. Clearly, group 2 covers a wide ontogenetic range and ideally should be divided into later childhood and adolescence [62] in order to better detail skeletal morphological changes. However, in terms of shape there is a degree of overlap between younger and older members of group2. Larger samples are necessary to investigate size and shape changes in more detail.

A relative increase in middle to lower thoracic width and depth (Figure 6e,k) could reflect previous findings [11] of deepening and an increase in posterior width in males between the ages of 20 and 65. Our data show that this is particularly so in the mid to lower ribcage and suggest that this not related to modifications of rib-shape itself but rather to modifications of sub-thoracic organs and modifications of the lower spine. Also, age-related shortening of pre-lumbar vertebral bodies and intervertebral discs could contribute to these changes.

One important critical aspect is that our data come from CT data sets produced in the supine position. As confirmed by a recent study variation in posture can affect functional performance [63]. To what extent changes in performance are related to posture-related changes in skeletal ribcage morphology should be addressed in future research.

Our results have shown that adult thoracic shape is the result of a shape trajectory that changes over time, it is curved. This reflects a modular growth pattern; the upper and lower parts of the thorax grow differentially because of differing relative influences of the developing lungs, abdominal viscera and the locomotor apparatus. Different kinematic factors are also likely important. For example, Ward et al [64] have suggested that muscle insertions divide the thorax into an upper compartment related to the insertions of the parasternal and scalenus muscle insertions (ribs 1-6) and a lower compartment related to the insertion of the diaphragm (ribs 7-12). This differential effect of muscles may reflect and point to the mechanisms underlying the mosaic evolution of upper and lower parts of the australopithecine thorax [19]. The differential growth of upper and lower thorax, quantified here, may be also be important in relation to understanding how childhood respiratory disease impacts on the development of adult thoracic shape and so function. However, better understanding of variations in thoracic form among recent and fossil humans as well as how these impact on function requires more extensive data and will be the subject of future studies.

## Supporting Information

Movie S1 **Ontogenetic allometry.** The movie shows the shape changes of the skeletal thorax represented by Principal Component 1 of Form space which reflects the major part of ontogenetic allometry. Note the different patterns of shape change in the upper and lower thorax units.
(AVI)
